# Patient-focused measures of functional health status and health-related quality of life in pediatric orthopedics: A case study in measurement selection

**DOI:** 10.1186/1477-7525-3-3

**Published:** 2005-01-12

**Authors:** William Furlong, Ronald D Barr, David Feeny, Suzanne Yandow

**Affiliations:** 1 Centre for Health Economics and Policy Analysis, and Department of Clinical Epidemiology and Biostatistics, McMaster University, Hamilton Canada; 2 Health Utilities Inc., Dundas Canada; 3 Department of Pediatrics, McMaster University, Hamilton Canada; 4 McMaster Children's Hospital, Hamilton Health Sciences, Hamilton Canada; 5 Institute of Health Economics, Edmonton Canada; 6 Departments of Public Health Sciences and Economics and Faculty of Pharmacy & Pharmaceutical Sciences, University of Alberta, Edmonton Canada; 7 Honolulu Shriners Hospital for Children, Honolulu, Hawaii USA

**Keywords:** health status, health-related quality of life, neurofibromatosis, orthopedics, pediatrics

## Abstract

The objectives of this report are to review the assessment of patient-focused outcomes in pediatric orthopedic surgery, to describe a framework for identifying appropriate sets of measures, and to illustrate an application of the framework to a challenging orthopedic problem.

A detailed framework of study design and measurement factors is described. The factors are important for selecting appropriate instruments to measure health status and health-related quality of life (HRQL) in a particular context. A study to evaluate treatment alternatives for patients with neurofibromatosis type 1 and congenital tibial dysplasia (NF1-CTD) provides a rich illustration of the application of the framework. The application involves great variability in the instrument selection factors. Furthermore, these patients and their supportive caregivers face numerous complex health challenges with long-term implications for HRQL.

Detailed summaries of important generic preference-based multi-attribute measurement systems, pediatric health profile instruments, and pediatric orthopedic-specific instruments are presented. Age-appropriate generic and specific measures are identified for study of NF1-CTD patients. Selected measures include the Activities Scale for Children, Gillette Functional Assessment Questionnaire Walking Scale, Health Utilities Index, and Pediatric Inventory of Quality of Life.

Reliable and valid measures for application to pediatric orthopedics are available. There are important differences among measures. The selected measures complement each other. The framework in this report provides a guide for selecting appropriate measures. Application of appropriate sets of measures will enhance the ability to describe the morbidity of pediatric orthopedic patients and to assess the effectiveness of alternative clinical interventions. The framework for measurement of health status and HRQL from a patient perspective has relevance to many other areas of orthopedic practice.

## Review

Assessments of patient-focused health status and health-related quality of life (HRQL) are being recognized increasingly by clinicians, patient advocates, regulatory authorities, administrators and policy makers as primary measures of the need, efficacy, effectiveness and efficiency associated with health care services. These types of measures are commonly reported in the published literature of many health care disciplines although there are few published reports concerning orthopedic surgery. However, the orthopedic community is becoming interested in using evidence reported by patients and patients' family members to determine what is best for patients.

Patient-focused evidence (including patient-reported outcomes, PROs) refers to results from functional health status (FHS) and HRQL studies of orthopedic outcomes with measurements obtained from the perspective of patients or their non-clinician caregivers (e.g., parents of patients too young to self-report). The field of orthopedics is concerned with all musculo-skeletal problems from the top to the bottom of the human body. This broad anatomical range is associated with a wide range of functional problems. Some orthopedic problems are highly focused in regards to anatomy and associated with highly effective therapies. However, other orthopedic problems are of a diverse nature, the etiology is often poorly understood, and effective treatment strategies are sometimes elusive.

The breadth and depth of some particularly challenging orthopedic problems has stimulated an interest in patient-based perspectives of their own FHS and HRQL. These types of measures will be referred to as "health measures" for the purposes of this paper. Health measures can be used to assess the burden of morbidity associated with orthopedic diagnoses, and to assess the effectiveness and efficiency of therapies. The diversity of issues also led the American Academy of Orthopedic Surgeons and the Pediatric Orthopedic Society of North America to develop the Pediatric Outcomes Data Collection Instrument (PODCI), and led other researchers to develop function-specific instruments such as the Activities Scale for Kids (ASK). However, there are a large variety of potentially appropriate health measures and there are limits to the number of these measures that can be used in any given study. The most appropriate set of health measures should be selected on the basis of underlying study objectives and design criteria [1].

Objectives and designs vary greatly across orthopedic studies. In general, orthopedic procedures and programs are undertaken to improve the health of patients. The health of patients can be measured in different ways. Conventional clinical or physiological measures are generally very useful for diagnostic and therapeutic purposes. Conventional clinical measures may, however, provide an incomplete assessment of health. For example, clinical examination and gait measures have been used to describe the quality and quantity of limitations in walking ability associated with hip flexion contracture but these types of assessments provide little information about the importance of this contracture relative to that of other serious health problems. In addition, various measures may provide conflicting results: some indicating improvement while others suggest decline in patients' health. Therefore, in the interest of scientific rigor, protocols to assess the overall effectiveness of therapy should include *a priori *specification of the comprehensive measure of health and a credible assessment viewpoint for the purposes of primary analysis of study results. Secondary study objectives may involve use of data collected from other health measures or from other assessment perspectives.

FHS and HRQL measures are important for a variety of reasons that complement conventional clinical measures [2]. FHS measures provide descriptive information and HRQL measures add some type of valuation about the desirability of overall health status. Valuation may be based on preference-based scores or by including only items identified as important to patients. HRQL is a more comprehensive concept than FHS as noted in this leading definition.

"Health-related quality of life is the value assigned to duration of life as modified by the impairments, functional states, perceptions, and social opportunities that are influenced by disease, injury, treatment or policy" [3].

It is generally accepted that a goal of therapy is to make patients feel better [4] and this is supported by statements of leading policy analysts such as "The goal of health care is to protect, promote, and maintain the health status of people" [5] and "Since the ultimate goal of all health care is to improve, restore, and preserve HRQL" [6]. However, physiological measures may change without people feeling better and people may feel better without measurable change in physiological function. Furthermore there is often a need for trade-offs between treatment-related benefits and adverse side effects. There is an increasing awareness also that various stakeholders in clinical decisions often have differing opinions about disability and that the opinions of patients should count. Numerous valid and reliable questionnaires are now available to collect FHS and HRQL measurements from patients and their representatives. The measures may be used for discriminative (comparing groups at a point in time such as assessing the burden of illness), evaluative (assessing within-person change over time as in clinical trials), and predictive purposes (providing prognostic information) [7].

A series of studies from a randomized controlled clinical trial (RCT) and prospective cohort study of elective total hip arthroplasty are illustrative of these purposes. These examples, while not from a pediatric setting, are orthopedic and demonstrate how data can be used for a variety of purposes. In the RCT, Rorabeck and colleagues [8] undertook a discriminative analysis of a set of FHS and HRQL measures, and reported there were no differences in outcomes between cemented and non-cemented protheses. Evaluative analyses of data from the same trial have documented improvements according to six HRQL measures and the six-minute-walk test [9], and have shown important differences among results from four major generic HRQL measures [10]. In the prospective cohort study by Mahon et al [11], waiting time for surgery was inversely related to baseline WOMAC (Western Ontario and McMaster University Osteoarthritis Index) scores for patients at time of referral to an orthopedic surgeon.

There are many factors to consider when designing studies using patient-focused health measures for orthopedic patients. Among the most important factors are the study objectives, types of patients, the dimensions and FHS constructs associated with the health problem under study, the appropriateness of the questionnaire for the age range of the patients, the viewpoint of the assessors answering the questionnaire, the method of collecting questionnaire information (self-completion or interviewer-administration), the period of time respondents are asked to consider when answering the questionnaire (i.e., assessment recall period), and measurement properties of assessment tools. Measurement properties include content and construct validity, test-retest and inter-rater reliability, responsiveness, and practical limitations of data collection. It takes many years, and often decades, for credible and relevant evidence to be accumulated about the measurement properties of instruments. Instruments with well-established measurement properties should be used whenever possible. Two prime issues for assessments of children are the developmental stage of the subjects and the most appropriate respondent for questionnaires.

The methods section of the paper presents a framework of important factors for consideration in designing studies to assess FHS and HRQL for orthopedic patients. The results were generated by applying the framework in the context of developing a proposal for a comprehensive international study of patients with neurofibromatosis type 1 (NF1) and congenital tibial dysplasia (CTD). The NF1-CTD protocol provides a rich illustration because it encompasses great variability in the instrument selection factors, and these patients with their supportive caregivers face numerous complex health status challenges with long-term implications for HRQL. Furthermore, there is little information in the published literature about the comprehensive health status and HRQL of patients with NF1-CTD. The rationale and approach to studying FHS and HRQL from a patient perspective have relevance to many other areas of orthopedics.

## Methods: a framework for orthopedic applications

This section identifies specific study design and measurement instrument factors that should be considered in selecting measures for use in studies, and presents some background information for illustrating the application of these factors.

### Study design factors

Study design factors are specified in detailed protocols that clearly define the study objectives and conditions. Well-defined study objectives identify the study subjects, and type and number of measurements required. Study conditions describe practical issues including data collection sites, and budgets for time and resources.

### Variability in study objectives

A highly focused, single objective study may require relatively few instruments while a broad-based, multi-faceted study will require numerous measures. For example, a RCT of efficacy for an experimental technique to achieve union of bone compared to a conventional technique might specify patients' reports from a single well-established walking ability scale as the primary outcome measure. However, an economic evaluation of the cost-effectiveness and cost-utility of the experimental technique would require at least two instruments: the single well-established walking ability scale to estimate effectiveness for the numerator in the cost-effectiveness ratio; and a utility-based scale of overall HRQL to estimate quality-adjusted life years for the numerator in the cost-utility ratio.

### Age range of subjects

The age of study subjects affects the choice of measurement instruments and the method of collecting data. Each instrument is valid for a specific age range and the age range is quite small for many pediatric instruments. For example, developmental changes during childhood make it difficult for single instruments to be valid from infancy through adolescence. Many instruments are valid for one or more of the following age categories: infants, pre-school children, primary-school children, adolescents, young / middle-age adults, seniors and elderly. Inability to read well (e.g., young children or populations with high illiteracy rates), or see well (e.g., many elderly populations), or concentrate well (e.g., people on certain strong medications) inhibits use of self-complete questions. Well-designed interviewer-administered questionnaires pose clear, short, well-focused questions with readily understood and easily remembered response options (e.g., yes or no response options).

### Assessment perspective

It is becoming widely accepted in many disciplines that studies of health care programs and technologies should include measures of patients' perceptions of their FHS and HRQL [12]. Measurements of these types have been shown to vary by assessment viewpoint and, although many different viewpoints are valid, the patient perspective is considered to be one of the most important. It has been shown that children as young as 7 years can reliably complete interviewer-administered disease-specific and generic questionnaires about their own health [13].

### Type of data collection sites

The locations and cultural characteristics of the study population will determine the language and sometimes the choice of assessors. Relatively few instruments have been carefully translated and culturally-adapted to facilitate use in a large variety of communities. Some cultures accept a variety of assessment viewpoints (e.g., patient, family, nurse, physician, and other allied health professionals) while other cultures recognize only a physician viewpoint for health assessments.

### Mode of data collection

The literature suggests that there may be important effects due to mode of data collection [14] and, therefore, mode of administration should be standardized across subjects, assessors, and assessment points. In general, self-complete questionnaires tend to elicit reports of greater morbidity than interviewer-administered questionnaires. It is hypothesized that subjects may be less inhibited to report disabilities on self-complete questionnaires than to report disabilities to interviewers. The mode of data collection for a study may be determined by numerous factors other than the characteristics of study subjects mentioned above. A low budget study may rely on a mail survey, or distribution of questionnaires in a busy clinic setting, and use self-complete questionnaires. Alternatively, a study involving serial assessments may require follow-up data to be collected by telephone and therefore require use of questionnaires designed for interviewer-administration.

### Assessment recall period

FHS questionnaires should have well-specified assessment periods to help ensure that the subjects and researchers know the period of time covered by the responses. Assessment recall period refers to the period of time the assessor is asked to consider when answering the questionnaire. The period should match the objectives of the study. For instance, if a new surgical technique is thought to reduce the peri-operative burden of morbidity then frequent assessments with a brief recall period (24 hours) might be suitable. Standard assessment periods include the past 24 hours, the past 1 week, the past 2 weeks and the past 4 weeks. Short assessment periods should be used in studies of patients whose FHS varies over time and in studies involving serial assessments to ensure that there is no overlap in assessment times. Relatively long recall assessment durations may be used when it can be assumed patients' health status is fairly stable. For example, a four-week recall assessment period was used to measure the HRQL of patients in a randomized clinical trial and economic evaluation of two alternative treatment strategies for patients with knee osteoarthritis [15,16].

### Other factors

There are limits to the number and type of measures that respondents can be expected to complete without getting tired or frustrated, and/or that the study budget can afford in regards to both data collection and analysis. The evidence about the limits of respondent burden is sparse. All else being equal, studies involving serial assessments should expect to collect fewer measurements per assessment than single-assessment cross-sectional surveys. To maximize efficiency, instruments should be selected to provide complementary rather than overlapping information.

### Instrument factors

#### Types of measures

There are numerous definitions of FHS and HRQL. For the purposes of the paper, a FHS measure is defined as being descriptive in terms of functional ability and HRQL is defined as involving some form of valuation of that health status.

One published taxonomy [7] suggests that measures may be classified as specific or generic. Specific measures are focused on a specified health problem, disease, or age group of subjects. An example is the Western Ontario McMaster Osteoarthritis Index [17]. Specific measures designed for evaluative purposes are often able to detect small but clinically important differences among subjects and be responsive to small but clinically important intra-subject changes over time. Generic measures are applicable to a broad range of subjects, including a wide variety of clinical groups and general populations. There are 2 types of generic measures: generic health profile instruments such as the Rand-36 [18] and SF-36 [19]; and generic preference-based instruments. There are 2 types of generic preference-based instruments: direct measurement instruments, such as standard gamble [20]; and multi-attribute classification systems with preference-based scoring functions [21], such as the Health Utilities Index [22,23] and the Quality of Well-Being Scale [24,25]. A detailed description of direct measurement techniques is beyond the scope of this paper but provided in a recent review paper by Torrance and colleagues [20]. Direct preference measurement is generally not practical for application in most clinical studies, especially those involving very young children. Customized instrumentation, usually relying on administration by highly skilled interviewers, must be developed for each study. Further, measurement questions are cognitively demanding. Direct preference measurement will, therefore, not be considered further in this paper.

Each multi-attribute system includes a descriptive classification scheme to describe and assess health status, and a preference-based valuation system. HRQL scores for health states defined by multi-attribute systems are calculated from models fitted from directly measured preference measurements (see below). Multi-attribute systems provide descriptive information about comprehensive health status, and interval-scale preference scores of overall HRQL from a community perspective on a scale where 0.00 is the score for being dead and 1.00 is the score for being in perfect health. Several multi-attribute systems define negative scores of overall HRQL to represent preferences for states considered worse than being dead. A few systems include single-attribute preference-based scales of morbidity. Single-attribute morbidity scales are defined such that the least desirable level within an attribute (dimension of health status such as vision) has a score of 0.00 (blind) and the most desirable level has a score of 1.00. The community perspective is most widely recommended for technology assessment and reference case economic evaluation analyses [26-29]. Interval-scale properties, and a score of 0.00 for being dead, are important features of HRQL scales for integrating the effects of morbidity and mortality in descriptive studies and in cost-utility economic evaluations. Interval-scale preference scores of HRQL may be either utilities (e.g., Health Utilities Index Mark 3) or values (e.g., EQ-5D). Utility preference measures are based on von Neumann-Morgenstern utility theory, include an element of risk attitude, and are therefore appropriate for decision problems with uncertainty. Value scores are preferences measured under certainty. Details about differences between utilities and values, and about direct preference measurement, appear in recent papers by Torrance et al [20,30]. Uncertainty is an important factor in many orthopedic procedures and therefore utility scores are more appropriate than value scores in this context.

### Evidence of measurement properties: validity, reliability, and responsiveness

A valid measure is "sound and sufficient" [31]. There are many ways to assess validity of measures. Assessments of FHS and HRQL measures should consider at least six types of validity: face validity, content validity, construct validity, convergent validity, discriminative validity, and predictive validity [32]. Face validity requires that a measure appear on the surface to make sense in regards to being relevant and useful. Content validity requires that the measure include all important and relevant domains or dimensions of health status. Construct validity describes the extent to which a measure corresponds to theoretical concepts and convergent validity describes the association between related variables. Discriminant validity is a lack of correlation between dissimilar variables or groups. Predictive validity, one type of criterion validity, describes the relationship between current and future measurements.

A measure is reliable if it is sound and dependable [31]. Reliability is assessed by tests of repeatability or reproducibility. Reliability is often assessed in terms of agreement between intra-subject test-retest measurements and inter-assessor measurements [33].

Responsiveness is also referred to as sensitivity to change. It is an important feature for determining a measure's ability to detect effects of treatments or natural changes over time (e.g., due to the aging process). Husted and colleagues reviewed the literature and defined two major types of responsiveness: internal and external responsiveness [34]. Internal responsiveness describes the ability of a measure (instrument) to change and has been assessed using a variety of techniques including the magnitude of statistical significance tests (e.g., p < 0.05 versus p < 0.001), the mean change score divided by the standard deviation of scores at baseline (effect size), and a sensitivity coefficient calculated as the proportion of the variance in change scores due to treatment [32]. It has also been assessed as the ratio of the mean change in patients' scores and the pooled standard deviation of the mean change scores [35], and as the mean change score among those who changed divided by the standard deviation of change scores among stable patients [33]. External responsiveness is concerned with the relationship between change in a measurement and change in a reference measurement of health status. External responsiveness has been assessed using the receiver operating characteristic method, correlations (e.g., Pearson product moment correlation), and regression models. The minimum important difference (MID) is the smallest size of difference that is important from patients' or clinicians' perspectives. The MID between two measurements is a concept closely related to responsiveness when assessing change over time [36].

Ceiling and floor effects are undesirable properties that reduce the validity, reliability, and responsiveness of measures. A ceiling effect may occur when a large proportion of measurement observations are close to the upper bound of the measurement scale. A ceiling effect results in a positively skewed distribution of measurements, limited ability of the measure to discriminate among subjects at the upper end of the scale, and attenuated responsiveness to improvements in health in longitudinal studies. A floor effect may occur when a large proportion of measurement observations are close to the lower bound of the measurement scale. Floor effects create a negatively skewed distribution of measurements, limited ability of the measure to discriminate among subjects at the lower end of the scale, and decreased responsiveness to decrements in health in longitudinal studies. Many generic and specific measures of HRQL may be subject to ceiling effect problems in that they may not be able to describe patients or subjects with above average (supra-normal) health. Some measures are subject to floor effect problems. Some are subject to both. Typically floor effect problems are more serious in clinical studies (which often involve patients with disabilities) and ceiling effect problems may be more problematic in general population studies.

### Limits of respondent burden

The limits of respondent burden depend upon many factors including the number of questions presented, how the questions are presented, the complexity of questions, the sophistication of respondents, and the respondents' interest in the questions. In general, the allowable length of questionnaire is shorter for mail and phone administration than for face-to-face interviewer administration [37]. One set of guidelines specifies the following maximum lengths: 20 questions for phone surveys; 60 questions for mailed surveys; and 80 questions for face-to-face interview surveys [38]. Another guideline recommends that telephone interviews not exceed 5 to 10 minutes [39]. These guidelines are in general agreement with maximum recommended number of pages for self-administered questionnaires: 2 to 4 page upper limit for topics not especially salient [40]; 12 page upper limit for self-administered questionnaires [41]; and 4 to 6 page upper limit for mailed surveys [42]. For mail-out surveys, the evidence suggests no effect of length on response rates for questionnaires varying from 3 to 9 pages [43,44] but reduced response rates with questionnaires greater than 12 pages [41].

### Availability of support services

Applications of FHS and HRQL measures are greatly facilitated by expert advice, detailed instructions and other services designed to support users of a measure. Supporting documentation is usually protected by copyright and should not be used without written permission of the original developers. Documentation obtained from third-party sources should be considered suspect because it is frequently invalid. Licensing fees are used to fund high quality, readily accessible service centers. Permission to use copyright materials is typically granted one study at a time. Support services may also include consultation about the most appropriate versions of questionnaires for use in a specific study. Application packages may include data collection instruments such as questionnaires, procedure manuals, coding algorithms and scoring systems, as well as background information about the conceptual and measurement properties of the instrument.

### NF1-CTD: A case study

Recently there has been interest in using measures of FHS and HRQL to evaluate treatment alternatives for NF1-CTD. NF1 is one of the most common genetic disorders in childhood [45]. It is estimated that at least 1 million people throughout the world have NF1 [46]. NF1 has a wide range of clinical manifestations including abnormalities of the skin, nervous system, bones and soft tissues [46]. Other conditions experienced by children with NF1 include short stature and neurologic problems such as learning disabilities or unspecified school performance problems (36%), frequent headaches (28%), mental retardation (6%), and reduced reproductive potential [46-49].

CTD is rare in the general population, approximately 1 per 140,000 [46]. It has been estimated that approximately 1% of people with NF1 have CTD [46]. CTD is diagnosed usually during the first year of life and fractures often occur before 3 years of age. Frequently, initial presentation is tibial bowing followed by subsequent fracture and pseudoarthrosis [45]. There is no generally accepted standard for management of CTD although most surgeons would suggest initial treatment of either intramedullary fixation with bone grafting or resection and bone transplant. Surgical procedures for the treatment of CTD are fraught with complications and failure of union. For the treatment of CTD, pre-fracture bracing until skeletal maturity may be a better alternative than surgery. CTD is associated with severe complications due to nonunion or pseudoarthrosis after osteotomy and amputation may be required.

Conventional clinical measures of CTD include the Crawford classification system [46]. These measures provide clinicians with important information used in diagnosis and management of well-established symptoms. A list of important concerns could be prepared by interviewing patients and members of their families. Standardized comprehensive tools that integrate multi-dimensional effects would also be useful in quantifying the number and extent of problems experienced by NF1-CTD patients, and other pediatric orthopedic patients with complex issues. The published literature on NF1 and NF1-CTD contains virtually no information based on FHS or HRQL measurements. The exception is a recent paper by Wolkenstein and colleagues [50] who reported results from 128 adult patients in France using the generic health profile SF-36 and a skin-disease-specific measure, Skindex-France.

Surveys of the published literature, experts in the fields, web sites and other sources of information were conducted to determine the dimensions of health that are affected by NF1-CTD, the types of FHS and HRQL measures that have been used, which measures should be considered as potentially useful for studies of NF1-CTD, the measurement properties of potentially useful measures, and the relative merits of various measures. A review of the on-line Quality of Life Instruments Database (QOLID) developed by Dr. Marcello Tamburini and the MAPI Research Institute [51], and correspondence with instrument developers, identified a short list of potentially useful measurement tools in each of the following categories: generic preference-based HRQL systems, major pediatric and other generic health profiles, and disease or function specific measures. Selected measures should have demonstrated properties in accordance with currently accepted criteria [12,52,53] and should provide commensurate measurements for patients across a wide age range. Problems with mobility, cognition, pain, emotion (including impacts of problems with self-image), self-care, vision, and fertility are aspects of health reported in the published literature to be compromised in NF1 patients.

### Illustrative study design criteria

There are five important research objectives of an NF1-CTD study that provide a context for applying the framework described in the Methods section:

1) to document long-term health outcomes associated with the disease and its treatment;

2) to measure the burden of disease and treatment during active therapy;

3) to investigate the hypothesis that improved HRQL is associated with initial amputation compared with multiple limb-saving procedures;

4) to determine relationships of FHS and HRQL with conventional clinical variables used in diagnosis and management; and

5) to assess the measurement properties (e.g., construct validity, patient versus parent inter-rater reliability, and responsiveness to change) of selected FHS and HRQL measures in NF1-CTD patients.

These detailed objectives require the identification and assessment of leading FHS and HRQL measures for use in both cross-sectional and prospective longitudinal surveys.

The prevalence of NF1-CTD is relatively low. Patients will need to be recruited from numerous clinical centers in North America to generate precise estimates of FHS and HRQL. Questionnaires should be available in at least 3 major languages: English, French and Spanish. The survey population ranges in age from newborn into adulthood and linking results across the study objectives requires that at least some of the assessment tools be in common across the age range of study patients. To avoid potential confounding effects, data collection techniques should be consistent across subjects and measures.

The patient-focus will be represented by collecting measurements from all patients old enough to provide self-assessments, and from parents acting as proxy assessors for all children and adolescents. Self-complete questionnaires requiring minimal supervision should be used to eliminate the need for interviewers at each clinical center, to facilitate use of mail-out surveys, and to avoid potential "interviewer" effects. The number and type of measures per assessment, and the number of serial assessments per patient, should be sufficient to address all the study objectives within the limits of study resources and assessor burden. Measures of morbidity associated with NF1-CTD should be comparable with data on norms from surveys of general populations and other patient groups, and be useful for assessing the effectiveness and efficiency of health care services.

### Existing patient-focused health measures

The HRQL measure should be comprehensive and preference-based, to facilitate a broad variety of comparisons. A pediatric health profile measure and other specific measures will be selected to complement the selected preference-based HRQL measure. FHS measures may be focused on one or more of the following: the population of interest (e.g., pediatrics); the major underlying disease (e.g., NF1); the major human function of most interest (e.g., walking ability); the medically-defined health problem of most interest (e.g., tibial dysplasia); the medical speciality most involved with treatment of the health problem (e.g., pediatric orthopedics).

There are six major generic preference-based HRQL utility systems [21], presented here in chronological order of development: QWB [25], 15D [54], HUI [23], EQ-5D [55], AQOL [56] and SF-6D [57,58]. HRQL scores from these systems represent mean community preference scores. The 15D and AQOL have not been widely used outside of Finland and Australia respectively and, therefore, will not be described further in this paper. The SF-6D has been developed only recently so there is as yet little evidence to report. The major features of QWB, HUI, EQ-5D, and SF-6D systems are summarized in Table 1[21,25,57,59,60]. The major characteristics vary greatly among the systems. For example, linear additive scoring models do not include effects of preference interactions among attributes or domains but multiplicative scoring functions include these effects. The QWB is available in both self-complete and interviewer-administered formats [61]. The symptoms attribute is a dominant feature of the QWB health status classification system. This emphasis is reflected in the population-derived preference weights. HUI health status classification systems cover more than 10 attributes. There is evidence that HUI scores agree well with mean directly measured standard gamble utility scores from a representative sample of the general population [59,60,62,63]. Numerous versions of HUI questionnaires are available and HUI has a service center [64-66]. It is available in numerous languages. A closely-related comprehensive health status classification system for pre-school children (CHSCS-PS) has been developed recently [67-69] for children age 2 through 5 years of age. EQ-5D is very simple and concise. It consists of 5 attributes with 3 levels per attribute, assesses "current" health status, has been used in a large number of studies, and is available in numerous languages. Information, including a long list of references, about EQ-5D is available on the EuroQol Group web site [70]. SF-6D is a multi-attribute health status classification system based on the SF-36 [19,71,72]. The SF-36 was not designed to be commensurate with the fitting of a multi-attribute utility function. The SF-6D health status classification system is a sub-set of the attributes defined in the SF-36 health status classification system [57]. SF-6D utility scores may be useful in retrospective studies analyzing previously collected SF-36 data.

**Table 1:** Major Characteristics of Five Generic Preference-Based Multi-Attribute Systems

**Instrument**	**QWB**	**HUI**		**EQ-5D**	**SF-6D**
				
		**HUI2**	**HUI3**		
**Developed**	1970s	1980s	1990s	1990s	1990s
**# Attributes (# levels per attribute)**	4 (3 to 27)	7 (3 to 5)	8 (5 or 6)	5 (3)	6* (4 to 6)
**# of unique health states**	1215	24000	972000	243	18000
**Attributes**	Mobility, Physical activity, Social activity, Symptoms / problems.	Sensation, Mobility, Emotion, Cognition, Self-care, Pain, Fertility.	Vision, Hearing, Speech, Ambulation, Dexterity, Emotion, Cognition, Pain.	Mobility, Self-care, Usual activities, Pain / discomfort, Anxiety / depression.	Physical functioning, Role limitations, Social functioning, Pain, Mental health, Vitality.
**Types of HRQL scores and measurement**	Values; VAS	Utilities; VAS and SG combination	Utilities; VAS and SG combination	Values; TTO	Utilities; SG
**HRQL scoring model form**	Linear additive	Multiplicative	Multiplicative	Modified linear additive	Modified linear additive
**HRQL scale interval**	0.00 to 1.00	-0.03 to 1.00	-0.36 to 1.00	-0.59 to 1.00	0.00 to 1.00
**Questionnaire formats (languages)**	2 (numerous)	16 (numerous)		1 (numerous)	2 SF-36 versions (numerous)

SG – standard gamble; TTO – time trade-off; VAS – visual analog scale.

* – 6 attributes but role limitations attribute above includes emotional and physical so encompasses 7 of 8 SF-36 domains.

The major population-specific health profiles include the Child Health Questionnaire (CHQ), Pediatric Inventory of Quality of Life (PedsQL), Pediatric Evaluation of Disability Inventory (PEI) and TNO-AZL Pre-School Children Quality of Life questionnaire (TAPQOL). The PEI is limited to children age 0.5 – 7 years of age and requires a structured parent interview or clinician observation [73]. TAPQOL [74,51] is limited to children 0.5 to 5 years of age. Therefore, PEI and TAPQOL will not be discussed further.

The major pediatric disease-/function-/specialty-specific instruments include the PODCI (also referred to as the POSNA or Pediatric Orthopedic Society of North America instrument), ASK, Gillette Functional Assessment Questionnaire Walking Scale (FAQ walking scale), and Wee-FIM. Wee-FIM [75], a popular measure of functional independence, is not being considered because it involves clinician assessments rather than assessments from a patient or parent perspective. In general, disease-specific scales in orthopedics focus on pain and physical function because these factors are major areas of concern for orthopedic patients and no generic health measures have been developed specifically for orthopedic application [73]. No relevant disease-specific measures or disease-specific preference-based tools were identified.

A summary of the major pediatric generic health profiles appears in Tables 2 and 3. The CHQ [76] covers relevant physical domains and provides detail on emotion/psychological health. The PedsQL [77] assesses physical, emotional, social and school functioning. It has demonstrated a return to health 3 months after acute limb fractures [78] and has been used in large general population surveys [79].

**Table 2:** Major Characteristics of Two Pediatric Health Profile Systems

**Instrument**	**CHQ**			**PedsQL 4.0**	
**Full Name**	Child Health Questionnaire			Pediatric Inventory of Quality of Life	

**Origin**	1996			1998	

**Version**	Parent Forms (PF)	Parent Form for Infants & Toddlers	Youth- Completion Form	Child Self-Report Forms 1) ages 5–7 years 2) ages 8–12 years 3) ages 13–18 years	Parent Report Forms 1) ages 2–4 years 2) ages 5–7 years 3) ages 8–12 years 4) ages 13–18 years

**Measurement Constructs**	Measures of physical and psycho-social health concepts			Measures of core dimensions delineated by WHO	

**Age Bounds (years)**	5 to 17	2 months to 5 years	11 to 17	5 to 18	2 to 18

**# Items**	98 (PF98) or 50 (PF50) or 28 (PF28) short form	102	87 (working on shorter form)	23	

**# Domains**	10 child & 4 parent concepts	8 infant & 5 parent concepts	10 child concepts	4 "Generic Core"	

**Overall Score**	2 Global Physical & Global Psycho-Social			1 overall, for primary analyses	

**Sub-scale Scores**	14	13	10	4 sub-scales, for secondary and descriptive analyses	

**Completion Time (minutes)**	10 to 20 for PF50; 7 to 15 for PF28	20 to 30	15 to 30	n/a	< 4

**Spanish Language**	Yes			Yes	

**Assessment Perspective**	Parents'	Parents'	Patients'	Patients'	Parents'

Legend: WHO – World Health Organization.

**Table 3:** Domains and Constructs of Forms for Two Pediatric Health Profile Systems

**Instrument**	**CHQ: Child Health Questionnaire**			**PedsQL 4.0**	
**Version**	Parent Forms (PF)	Youth-Completion Form	Parent Form for Infants & Toddlers	Child Forms (3 versions: 5–7, 8–12, 13–18 yrs)	Parent Forms (4 versions: 2–4, 5–7, 8–12, 13–18 yrs)

**Domain Names**	1. Physical functioning – 6 items. - e.g., Child is greatly limited in performing all physical activities, including self-care, due to physical health. 2. Role (school and activities) limitations due to emotional.* 3. Role limitations due to behavioral problems* – 3 items. - e.g., Child is limited a lot in school work or activities with friends due to behavioral problems. 4. Role limitations due to physical problems – 2 items. - e.g., Child is greatly limited in school work or activities with friends due to physical health. 5. Bodily pain – 2 items. - e.g., Child has extremely severe, frequent and limiting body pain. 6. General behavior – 6 items. - e.g., Child very often exhibits aggressive, immature, delinquent behavior. 7. Mental health – 5 items. - e.g., Child has feelings of anxiety and depression all the time. 8. Self-esteem – 6 items. - e.g., Child is very dissatisfied with abilities, looks, family/peer relationships and life overall. 9. General health perceptions – 6 items. - e.g., Parent believes child's health is poor and likely to get worse. 10.Change in health – 1 item. - e.g., Child's health is much worse now than 1 year ago.		1. Physical Functioning. 2. Behavior (perception). 3. Growth & Development. 4. Pain. 5. Behavior (getting along). 6. Temperament & Mood. 7. General Health. 8. Change in Health.	1.. Physical Functioning – 8 items. - hard to walk more than one block, hard to run, hard to do sports or exercises, hard to lift something heavy, hard to take a bath, hard to do chores around the house, hurt or ache, low energy. 2. Emotional Functioning – 5 items. - feel afraid or scared, feel sad or blue, feel angry, trouble sleeping, worry about what will happen. 3. Social Functioning – 5 items. - trouble getting around, other kids not wanting to be friends, teased, doing things other peers do, hard to keep up when playing with others. 4. School Functioning – 5 items. - hard to concentrate, forget things, trouble keeping up with schoolwork, miss school because not well, miss school for doctor appointments.	

*Role Emotional and Role Behavioral are separate scales in PF98 but combined into one in the PF50 and PF28.

Table 4 summarizes the major characteristics of four orthopedic-specific measures. The PODCI [80] was designed specifically as a very comprehensive measure of musclo-skeletal outcomes associated with pediatric orthopedic problems. ASK was designed to measure children's activities in terms of both capacity and performance [81], and it assesses domains not covered in detail by other instruments [82]. The FAQ walking scale provides the most complete measure of walking abilities.

**Table 4:** Major Characteristics of Orthopedic-Specific Systems

**Instrument**	**PODCI**	**ASK**	**FAQ walking scale**
**Full Name**	Pediatric Outcomes Data Collection Instrument	Activities Scale for Kids	Gillette Functional Assessment Questionnaire: Functional Walking Scale

**Origin**	1997	1996	1993

**Measurement constructs**	Musculoskeletal outcomes	Physical disability in terms of capacity or performance dimensions	Complete range of functional walking abilities

**Age range (years)**	2 – 18	2 – 18	2 +

**# Items**	108	30	1

**# Domains; and Domains**	8 Comorbidity, Upper Extremity & Physical Function, Transfers & Basic Mobility, Sports & Physical Function, Pain, Treatment Expectations, Happiness, Satisfaction with Symptoms.	9 Personal Care, Dressing, Eating & Drinking, Miscellaneous, Play, Locomotion, Standing Skills, Stairs, Transfers.	1 Walking.

**Overall Score**	Yes, Global Function, mean of sub-scales for domains 1 to 4 above	Yes, additive and not weighted	Yes

**Sub-scale Scores**	Yes, additive	No	No

**Completion Time (minutes)**	10 – 20	5 – 12	< 2

**Spanish Language version**	No	No	No

**Assessment Perspective**	Patient &/or Parent (Diagnostic, complications and aims section by clinician)	Patient	Parent

The choice of existing measures is based on a process of elimination considering the relative strengths of each instrument and the complementarities among measures. Neither the SF-36 nor the EQ-5D is valid for use in adolescent patients with orthopedic problems [83]. A review of measurement of HRQL in children by Eiser & Morse [[84]; see also [85,86]] identified HUI and CHQ and PedsQL as the only 3 generic measures that fulfill all specified review criteria: established reliability and validity; suitable for self- and proxy-report; and brief (<30 items). PODCI outperformed CHQ physical functioning scale for orthopedic patients [87]. However, PODCI has considerable problems with missing data, especially in upper extremity function and physical function and sports scales for children ages 2 to 5 years, associated with the use of "too young" response options [88]. ASK is reported to be more sensitive to change in disability levels than HUI [Young N, personal email communication to W Furlong 2002-02-18]. The FAQ walking scale provides the most complete assessment of functional walking abilities, especially at the upper end of the scale [89].

In summary, there are few measures available for assessing subjects less than 5 years of age and even fewer for subjects less than 2 years of age. Most relevant measures are available in self-complete format only. Preliminary recommendations for the NF1-CTD study were that PedsQL be used as the generic health profile, HUI be the multi-attribute preference-based measure of HRQL utility scores for children age 5 years and older and that CHSCS-PS be the measure for children age 2 through 4 years, ASK be the measure of activity limitation, FAQ walking scale be the measure of walking ability, and that a small feasibility study of these instruments be completed with a convenience sample.

### Feasibility study

A pilot feasibility study surveyed 8 NF1 patients using HUI and FAQ walking scale measures. Questionnaires were completed by 6 NF1 patients and 3 parents. The combined HUI and FAQ walking scale questions took respondents an average of 13 minutes (range, 9–20 minutes) to complete. The patients were 11 to 50+ years old and had health problems ranging from mild to severe. One patient with tibial dysplasia and 2 patients with scoliosis were included.

HUI data were collected from 5 patients, 2 parents and both the patient and parents in one case. Health problems were reported in 7 of the 8 HUI3 attributes (vision, speech, ambulation, dexterity, emotion, cognition, and pain; no problems with hearing were reported). The attributes associated with the most morbidity, as assessed using HUI3 single-attribute utility scores [60], were pain (mean score = 0.81), speech (0.94), cognition (0.94), and emotion (0.94). For the 7 patients having complete data, 5 had two or more HUI2 and HUI3 attributes at less than full function. On the conventional utility scale in which being dead = 0.00 and in perfect health = 1.00, the HUI3 scores ranged from 0.45 to 1.00. The mean HUI3 score, 0.73, is similar to the mean score of 0.77 for adults with arthritis [90].

FAQ walking scale data were collected from 4 patients (1 of the 5 survey patients did not answer the question), 2 parents and both the patient and parents in one case. Five patients were reported to be at Level 10 (walks, runs, and climbs on level and uneven terrain and does stairs without difficulty or assistance), one patient to be at Level 8 (walks outside the home for community distances, is able to get around on curbs and uneven terrain in addition to level surfaces, but usually requires minimal assistance or supervision for safety), and one patient at Level 6 (walks more than 15–50 ft. outside the home but usually uses a wheelchair or stroller for community distances or in congested areas).

In summary, the feasibility study showed that the HUI and FAQ walking scale questions were acceptable to patients' families and that results, especially for HUI, reflected the large variability in HRQL of the sample of patients.

### Choice of measures for illustrative study

No single measure will provide sufficient data to address all the important study objectives. A set of measures is required. The set of measures should provide complementary data of health status and preference-based scores of HRQL. Redundancy in measurement is reduced, and efficiency of measurement is increased, by selecting the most comprehensive generic measures and then supplementing these measures with the most appropriate set of specific measures.

It is recommended that HUI be selected as the comprehensive generic measure for ten reasons:

a) it includes both generic health profile and preference-based scoring systems;

b) the preference-based scoring systems are well-validated;

c) it is the most comprehensive, compact and efficient of these types of systems;

d) it includes many of the most important domains in the context of NF1-CTD;

e) it is applicable for all people age 5 years and older;

f) well-developed data collection questionnaires are available to match the study design criteria;

g) HUI results facilitate integrating effects of morbidity and mortality, and cost-utility economic evaluations;

h) it has been used successfully in a variety of studies of musculoskeletal problems;

i) population norm data are available; and

j) a closely-related health status system, the CHSCS-PS, is available to assess children 2 through 4 years of age.

The HUI will provide a broad set of measures for comparisons with other populations and for estimating HRQL on a general scale such that dead = 0.00 and perfect health = 1.00. As a generic measure, HUI also has the ability to capture side effects and the effects of co-morbidities. However, these broad measures may not be responsive to small but important changes in health status. Therefore, HUI should be complemented by a set of instruments focused on pediatric, orthopedic and walking issues.

The PedsQL 4.0, a pediatric generic health profile, should also be included in the set of measures because:

a) it includes domains, social and school function, which complement HUI and CHSCS-PS domains;

b) it is appropriate for children ages 2 through 18 years;

c) it is not overly burdensome in terms of data collection;

d) patient and parent assessment questionnaires are available; and

e) it can be interviewer-administered to facilitate data collection by telephone, if necessary.

Two specific measures should also be part of the set of instrumentation: the ASK and the FAQ walking scale. ASK is an orthopedic-specific instrument which has been shown to cover the most important domains in the context of musculoskeletal disorders, including the impact of limb lengthening surgery experienced by many children with tibial dysplasia. ASK is also attractive because it provides overall summary scores for both performance and capability measures, and is only moderately burdensome to complete. Walking ability is one of the most important aspects of health that is frequently compromised in NF1-CTD patients, and the FAQ walking scale is the most complete scale of functional walking ability currently available and it only requires asking one question.

CHQ is not recommended because it does not add much to the set of recommended measures and it is burdensome to complete. PODCI is not recommended because it is very burdensome to complete, it has been reported to have major problems with "missing data", and the system for collapsing questionnaire responses into summary scores is not well validated.

Children ages 2 to 5 years of age should be assessed by their parents using three questionnaires: the CHSCS-PS (12 questions), the PedsQL (23 questions); and the FAQ walking scale (1 question). It is expected that all three of these questionnaires can be completed in an average of 15 minutes.

Children and adolescents ages 5 to 17 years of age should be assessed by their parents using four questionnaires: the HUI (15 questions), the PedsQL (23 questions), the FAQ walking scale (1 question), and the ASK (30 questions). These four questionnaires are expected to be completed in an average of 20 to 30 minutes.

Children and adolescents older than 11 years should provide self-assessments using four questionnaires: the HUI (15 questions), the PedsQL (23 questions), the FAQ walking scale (1 question), and the ASK (30 questions). On average, it is expected that respondents will complete all four questionnaires in 20 to 30 minutes.

## Conclusions

This paper highlights reasons why patient-focused measures of FHS and HRQL should be considered important tools in the field of orthopedic surgery. It has also noted that there is increasing competition for scarce health-care resources, that allocation decisions about these resources are being informed by evidence based on patient-focused health measures, and that these measures are being under-utilized by the orthopedic surgery community.

The orthopedic community faces numerous obstacles in utilizing FHS and HRQL measures. One major obstacle is that the multitude of existing measures makes it difficult to decide which measures may be appropriate for a specific application. A second obstacle is that most of the information about FHS and HRQL measures is not reported in the orthopedic literature. A third obstacle is that usually no one measure can capture all the important aspects associated with a specific orthopedic issue. The framework outlined in the paper provides guidance for selecting appropriate FHS and HRQL measures. The framework guides orthopedic investigators to combine their basic study criteria, including objectives and clinical context, with key criteria for FHS and HRQL measures from the published literature.

The results in this paper identify some major sources of information about health measures, identify some of the most widely used measures of FHS and HRQL, and provide summaries of key characteristics for selected measures in three major taxonomical classes: generic preference-based multi-attribute systems; generic pediatric health profile systems; and orthopedic-specific systems. It is clear that there are many important differences among measures both within and across taxonomical classes. All measures are not equal. There are sound factors for making judgements about which measures are most appropriate for a given application. A process of appraisal and elimination was used to select one measure from each taxonomical class for inclusion in the NF1-CTD study illustrative example, and a pilot study of the most readily available selected measures confirmed the feasibility of their use in a small sample of NF1-CTD patients.

The paper shows that a set of relevant, valid, reliable, responsive and practical patient-focused health measures for use in an orthopedic study can be readily identified and selected from the published literature and information available on the worldwide web. We encourage orthopedic researchers to use the framework to identify and select appropriate patient-focused health measures in their future studies.

## Conflict of Interest

W. Furlong and D. Feeny have a proprietary interest in Health Utilities Inc. which distributes copyright Health Utilities Index (HUI^®^) instrumentation and provides methodological advice on the use of HUI.

## Authors' contributions

All authors were involved in critical review of drafts for intellectual content and have given final approval to the version submitted for publication. W Furlong was responsible for much of the overall design, acquisition of data, and initial drafting. R Barr and D Feeny made important contributions to the analysis and interpretation of results. S Yandow conceived the idea of presenting the information in a published manuscript.
